# Assembly dynamics of PML nuclear bodies in living cells

**DOI:** 10.1186/1757-5036-3-3

**Published:** 2010-03-05

**Authors:** Peter Brand, Thorsten Lenser, Peter Hemmerich

**Affiliations:** 1 Leibniz-Institute of Age Research, Fritz-Lipman-Institute, Beutenbergstr, 11, 07745 Jena, Germany; 2 Institute of Computer Science, Friedrich-Schiller-University, 07743 Jena, Germany

## Abstract

The mammalian cell nucleus contains a variety of organelles or nuclear bodies which contribute to key nuclear functions. Promyelocytic leukemia nuclear bodies (PML NBs) are involved in the regulation of apoptosis, antiviral responses, the DNA damage response and chromatin structure, but their precise biochemical function in these nuclear pathways is unknown. One strategy to tackle this problem is to assess the biophysical properties of the component parts of these macromolecular assemblies in living cells. In this study we determined PML NB assembly dynamics by live cell imaging, combined with mathematical modeling. For the first time, dynamics of PML body formation were measured in cells lacking endogenous PML. We show that all six human nuclear PML isoforms are able to form nuclear bodies in PML negative cells. All isoforms exhibit individual exchange rates at NBs in PML positive cells but PML I, II, III and IV are static at nuclear bodies in PML negative cells, suggesting that these isoforms require additional protein partners for efficient exchange. PML V turns over at PML Nbs very slowly supporting the idea of a structural function for this isoform. We also demonstrate that SUMOylation of PML at Lysine positions K160 and/or K490 are required for nuclear body formation *in vivo*.We propose a model in which the isoform specific residence times of PML provide both, structural stability to function as a scaffold and flexibility to attract specific nuclear proteins for efficient biochemical reactions at the surface of nuclear bodies.

**MCS code:** 92C37

## 1 Background

The cell nucleus is functionally devoted to the realization and protection of the genetic material it contains in the form of chromosome territories [1]. RNA transcription and processing, DNA replication and DNA repair occur in a spatio-temporal coordinated fashion in small, usually less than 100 nm large foci scattered throughout the nuclear volume [2-4]. In addition, the mammalian cell nucleus contains a variety of internal structures, also termed domains or bodies [5]. These macromolecular assemblies include nucleoli, speckles, Cajal bodies, and promyelocytic leukemia nuclear bodies (PML NBs) [6,7]. While the structure and function of nucleoli, which is mainly ribosomal RNA synthesis and ribosome biogenesis, is very well understood, the precise biochemical function of speckles, Cajal bodies or PML nuclear bodies is not known [6]. With the exception, again, of the nucleolus which builds on ribosomal RNA genes, it also remains elusive if and how the other nuclear domains are spatially and functionally related to sites of transcritpion, replication, DNA repair, or how they relate to specific genomic regions [7].

PML nuclear bodies, also known as nuclear domain 10 (ND10) are macromolecular protein assemblies in the nucleus of mammalian cells. They have been implicated in key cellular functions including cell cycle progression, the DNA damage response, transcriptional regulation, viral infection, and apoptosis, however the precise biochemical functions of PML NBs in these processes is not known [8,9]. PML NBs range in size from 0.2 μm to 1.2 μm in diameter [10]. The number and distribution of PML NBs varies considerably depending on cell type, cell cycle and cell condition, but typically between 10 and 20 PML NBs can be found per nucleus [11]. Electron and 4Pi-microscopy revealed a ring-like shape of PML NBs under normal growth conditions with an 50 to 100 nm thick proteinacous outer shell [10]. The core of PML NBs was found either free of protein, DNA, or RNA accumulations [10-12], or to contain specific SUMO isoforms or specific chromatin subregions [10]. Chromatin threads and RNA in direct contact with the surface of the bodies might help to stabilize nuclear body structure [13,14].

**Figure 1:**
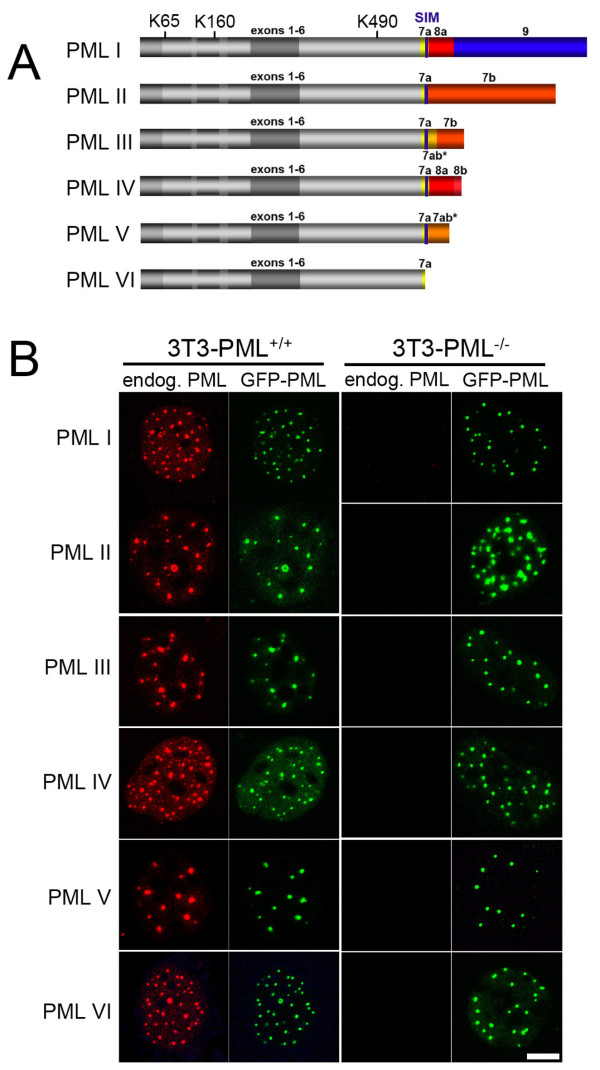
**All PML protein isoforms form nuclear bodies in cells lacking endogenous PML**. (A) Schematic depiction of the domain structure of PML isoforms (data taken from [16]). All PML isoforms share a common N terminus (exons 1 to 6) but differ in their C termini due to alternative spilicing of exons 7 to 9. Numbers indicate exons. Stars indicate retained intron sequences. The postion of three SUMO-modifyable Lysin (K) residues are indicated. Note that PML VI does not contain and the SUMO-interacting motiv (SIM) present in the other isoforms. (B) Mouse 3T3 cells with (3T3-PML^+/+^) or without (3T3-PML^-/-^) endogenous PML expression were transfested with expression vectors encoding human PML isoforms I to VI as GFP fusion proteins. Cells on coverslips were fixed and processed for immunofluorescence staining to detect endogenous mouse PML protein (red) and the exogenous GFP-tagged human PML isoforms (green). Images show mid-nuclear confocal sections. Note that the anti-mouse-PML antibody does not cross-react with human PML. Bar, 5 μm.

The signature protein of PML NBs is the promyelocytic leukemia gene product because PML-negative cells are unable to form nuclear bodies and other PML NB components show a dispersed nuclear distribution [15]. Six nuclear PML isoforms which vary in their carboxy termini are expressed by alternative splicing of the PML gene in humans (Fig. 1A) [16,17]. PML proteins may exert their isoform-specific functions through interaction with specific protein partners at nuclear bodies or within chromatin away from the bodies, or both [8]. Proteins present at PML NBs at endogenous expression levels include Sp100, Daxx, the Bloom's syndrome gene product (BLM), the small ubiquitin-related modifier 1-3 (SUMO1-3), and NDP 55 [18].

The formation of PML nuclear bodies relies primarily on the self-assembly abilities of the N-terminal RBCC domain in PML, and its SUMOylation status [15,19,20]. PML as well as other PML NB components, such as Sp100 and Daxx contain a SUMO interacting motif (SIM) with which these proteins can bind SUMO noncovalently [21]. Binding of proteins to PML nuclear bodies can therefore be modulated by noncovalent interactions between the SUMO moieties and SIMs of PML-interacting components [22,23].

PML nuclear bodies may be directly involved in biochemical reactions in the cell nucleus by modulating chromatin structure, regulating transcription of specific genes, sequestering of specific nuclear proteins, and/or mediating posttranslational modifications of specific target proteins [8]. Inherent to all these models is the question if PML NB components function directly within this structure or somewhere outside at different intra-nuclear sites, or both. A regulated network traffic between these sites may constitute a potential control mechanism with PML at its core, as suggested very early [24].

In order to reveal and study such mechanisms we have previously assessed the biophysical properties of PML nuclear body components and the assembly dynamics of these macromolecular domains in nuclei of living human cells using fluorescence correlation spectroscopy, fluorescence recovery after photobleaching and mathematic modeling [25]. These analyses uncovered a kinetic model for factor exchange at PML nuclear bodies and highlighted potential mechanisms to regulate intra-nuclear trafficking of PML NB components. To further characterize the assembly of PML nuclear bodies we now performed biophysical analyses in living mouse cells lacking endogenous PML proteins. This allowed us to study the nuclear body formation abilities of individual PML isoforms in a live cell setting.

## 2 Methods

### Cell Culture and Transfection

Mouse 3T3-PML^+/+ ^and 3T3-PML^-/- ^cells [26], kindly provided by T. Hofmann (DKFZ Heidelberg) were cultured in Dulbecco' modified Eagle's medium (DMEM) supplemented with 10% fetal calf serum in a 10% CO_2 _atmosphere at 37°C. For live cell imaging experiments, cells were seeded on 42 mm glass dishes (Saur Laborbedarf, Reutlingen, Germany) and transfected with plasmid DNA one to two days before observation using FuGENE-HD Transfection reagent (Roche, Basel, Switzerland) according to the manufacturer' protocol.

### Plasmids

The GFP-PML expression constructs have been described in detail previously [25].

### Western blots

Whole cell extracts were produced from transiently or stably transfected cell lines, electrophoresed on SDS-PAGE and transferred to Protran nitrocellulose membrane (Schleicher & Schuell, Dassel, Germany). The membrane was incubated with primary antibodies (in PBS-T) and developed with a peroxidase conjugated secondary species-specific antibody (Jackson Immunoresearch, West Grove, PA, USA). Signal was detected using the ECL reagent (Amersham, Uppsala, Sweden) on imaging film (Biomax, Kodak, Stuttgart, Germany). Anti-GFP monoclonal antibody was from Santa-Cruz Biotechnology (Heidelberg, Germany). Anti-mouse PML monoclonal antibody (# 05-718), non-cross reactive with human PML protein, was purchased from Upstate.

### Immunocytochemistry and Microscopy

Cells grown on 15 mm diameter coverslips were fixed with 4% formaldehyde for 10 minutes and permeabilized with 0.25% Triton-X100 for 3 minutes. Diluted anti-mouse PML mAB was incubated on cells for 45 minutes. After 3 washing steps with PBS, an anti-mouse secondary antibody coupled to Cy3 (Jackson Immunosearch, West Grove, USA) was incubated on cells for 45 minutes, followed by a DNA-staining step using ToPro3 or DAPI (Invitrogen, Carlsbad, USA) for 10 minutes and mounting with Prolong Gold antifade mounting medium (Invitrogen, Carlsbad, USA). For microscopy, a LSM 510Meta or LSM710 laser scanning confocal microscope (Carl Zeiss, Jena, Germany) was used.

### Fluorescence Correlation Spectroscopy Measurements

Fluorescence correlation spectroscopy (FCS) measurements were performed at 37°C on a LSM 510Meta/ConfoCor2 combi system using a C-Apochromat infinity-corrected 1.2 NA 40× water objective (Carl Zeiss, Jena, Germany) as described in detail elsewhere [25]. Briefly, GFP-tagged proteins were spot-illuminated with the 488 nm line of a 20 mW Argon laser at 5.5 Ampere tube current attenuated by an acousto-optical tunable filter (AOTF) to 0.1%. The detection pinhole had a diameter of 70 μm and emission was recorded through a 505-530 nm band-path filter. For the measurments, 10 × 30 time series of 10 s each were recorded with a time resolution of 1 μs and then superimposed for fitting to an anomalous diffusion model in three dimensions with triplett function [27] using Origin Software (OriginLab, Northhampton, MA, USA). The diffusion coefficients and anomaly parameters were extracted from fit curves as previously described [25].

### Fluorescence Recovery after Photobleaching

Fluorescence Recovery after Photobleaching (FRAP) experiments were carried out on a Zeiss LSM 510Meta confocal microscope (Carl Zeiss, Jena, Germany). One or two image stacks were taken before the bleach pulse and 50-70 image stacks after bleaching of "regions of interest" (ROIs) containing one nuclear body each at 0.05% laser transmission to minimize scan bleaching. Image aquisition frequency was adapted to the recovery rate of the respective GFP fusion protein, usually a 20 second interval was applied. The pinhole was adjusted to 1 airy unit. The image stacks were maximum-projected into a single plane from which relative fluorescence intensities within the ROIs were quantitated according to [25] using Excel (Microsoft, Redmond, WA, USA) and Origin software (OriginLab, Northhampton, MA, USA).

### Reaction-diffusion model

For the mathematical model, the structural complexity of a PML body has been approximated assuming that molecules undergoing binding and unbinding to and from the body do so at the surface, and molecules situated more towards the inside of the body cannot unbind before moving to the surface. There is thus a reservoir of tightly bound "inner" molecules and one of loosely bound "outer" ones. Exchange between these reservoirs is modeled by linear kinetics, i. e. the more molecules there are, the more will move inside or out with rate constants *k*_in _and *k*_out_, respectively. Binding and unbinding to the PML body is treated similarly, with rate constants *k*_on _and *k*_off_, respectively. The experimental set-up provided that bleach ROI and FRAP ROI is similar, and so are assumed to be the same for modelling purposes. Diffusion inside and out of the ROI was modelled as a linear two-way process with a rate constant proportional 2D/r^2^, where D is the diffusion coefficient measured by FCS, and r is the ROI's radius. This constant yields the effective exchange rate through the boundary of a circular area. The model considers only fluorescent molecules of one type at a time, so no interactions between different molecular species are considered. It distinguishes between molecules outside the ROI, inside the ROI but diffusing freely, loosely bound at the surface of the PML body, and tightly bound inside the body. The fluorescence outside the ROI is all but unchanged by the bleaching pulse and subsequent diffusion, so this value was used to normalize all concentrations in the equations. Describing normalized concentrations of the fluorescent protein in free diffusion (x), loosely bound (y) and tightly bound (z), the reaction system results in the model equations

The differential equations were numerically solved using an explicit Runge-Kutta formula (method ode45 in MATLAB). To fit the parameters of the model, an Evolution Strategy with Covariance Matrix Adaptation (CMA-ES) was employed [28].

We determined the ratio *p *= 20 of steady state fluorescence in the body vs. the background by confocal microscopy of GFP-PML isoforms and pixel intensity evaluation using MetaMorph software (Molecular Devices, Sunnyvale, USA).

This enabled us to express the observable fluorescence in the ROI as

Since the amount of bleached molecules is small compared to the overall amount of molecules in the nucleus, it is plausible to assume that after a sufficiently long time, fluorescence returns to the value measured before the photobleach. This constraint removes one degree of freedom from the model, yielding

These values were used to normalize the concentrations used in the model. The mathematical model treats normalized concentrations of fluorescent molecules in free diffusion (x), loosely bound (y) and tightly bound (z) to the PML body.

It is important to note that the concentration values x, y and z have been normalized by the background fluorescence outside the region of interest. Since we have determined the ratio *p *between equilibrium fluorescence inside the body and outside the ROI, and because the data used to fit the model was given in relative fluorescence intensity with an equilibrium value of 1, the background flourescence is given by 1/*p*. Therefore, the observed RFI value *w *is related to the model concentrations by

Over a very long time, all molecular species in the PML-body will eventually turn over, so that it is reasonable to assume *w*(t) tends towards 1 for large t. This means that for equilibrium conditions, in which , w(t) = 1. In this case, we have

and thus, from the first three lines, we get

Finally, taking this together with the conditition *x + y + z *= *p*, we find

This enabled us to remove one degree of freedom, *k*_on_, from the model.

## 3 Results and Discussion

### 3.1 All PML isoforms form nuclear bodies in PML-/- cells

We had previously analyzed the dynamics of component exchange at PML nuclear bodies in human cells expressing endogenous PML proteins [25]. The objective of the current study was to study the formation of PML NBs in the absence of endogenous PML expression. All six human PML isoforms (Fig. 1A) were therefore expressed as GFP fusion proteins in mouse 3T3 control cells (3T3-PML^+/+^) or mouse 3T3 cells derived from PML knock-out mice (3T3-PML^-/-^). All GFP-PML constructs are functional in human cells [25] and were expressed as full-length proteins in 3T3 cells, as judged by western-blotting (data not shown).

Immunofluorescence analyses showed that all six human GFP-PML isoforms localized to enogenous PML nuclear bodies in 3T3-PML^+/+ ^mouse cells (Fig. 1B). Importantly, in 3T3-PML^-/- ^mouse cells, each individual human GFP-PML isoform was able to form nuclear bodies (Fig. 1B). This confirmed that the nuclear body formation ability of PML resides within sequences encoded by exons 1 to 6, represented by GFP-PML VI, and suggests that the C-terminal extensions of PML isoforms (exons 7 to 9) do not alter this function (Fig. 1B). Because GFP-PML VI which does not contain the SUMO-interacting motiv (SIM) (Fig. 1A) is still able to form nuclear bodies in the absence of endogenous PML bodies we conclude that a SIM is not essential for nuclear body formation by PML as previously suggested [22].

### 3.2 PML isoform specific binding properties at nuclear bodies

**Figure 2:**
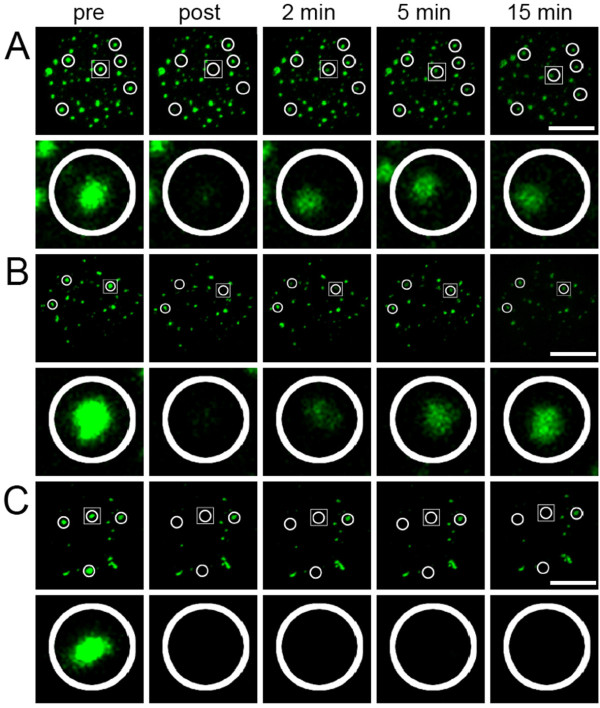
**FRAP to determine exchange of PML isoforms at nuclear bodies**. (A) FRAP experiment of GFP-tagged PML II in 3T3-PML^+/+ ^cells. Fluorescence was bleached in circled areas containing a nuclear body as indicated. Image stacks of the whole nucleus were taken before (pre) and after (post) the bleach pulse and at different time points thereafter. The upper row shows the projection of the whole nucleus while the bottom row shows an enlarged view of the bleached nuclear body indicated by a white box. (B and C) Same experiment as in (A) using 3T3-PML^-/- ^cells. In one cell population fluorescence recovery was observed with kinetics similar to PML wild type cells (B) while in another fraction of cells no recovery of GFP-PML II at nuclear bodies was oberved (C). Bar, 5 μm.

**Figure 3:**
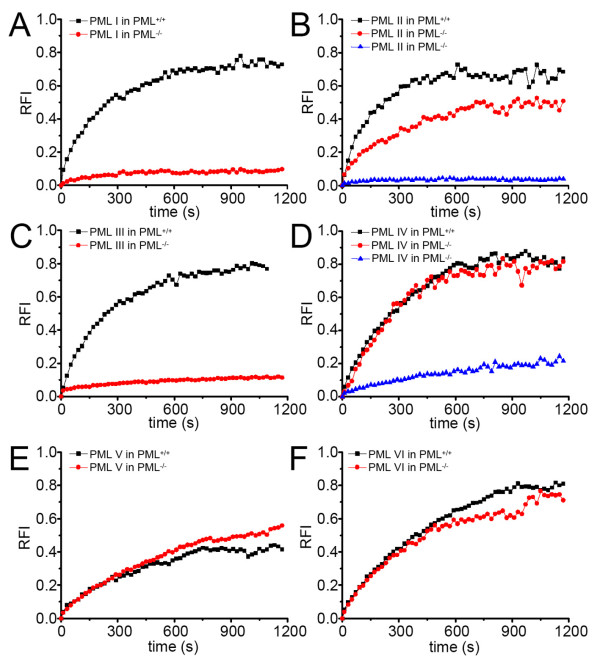
**Quantitation of FRAP experiments**. FRAP experiments as described in Figure 2 were performed for all GFP-PML isoforms in PML positive (black curves) and PML negative cells (red curves) as indicated. A second mobility population of PML negative cells was observed for GFP-PML II and IV (blue curves). The graphs show the mean values from at least 20 FRAP evaluations as relative fluorescence intensity (RFI) after normalization to prebleach levels. Standart deviations (not shown) ranged between 10 to 15 percent of the mean values.

To study the binding properties of individual PML isoforms at nuclear bodies we employed FRAP. A spherical region containing one PML nuclear body was bleached to background levels and fluorescence recovery was monitored for 20 min (Fig. 2). Similar to human cells [25], all six GFP-PML isoforms exhibited protein-specific FRAP curves in 3T3 cells expressing endogenous PML nuclear bodies (Fig. 3, black curves). In particular, we could confirm that exchange of GFP-PML V is extremely slow: fluorescence recovered to only ~40% after 20 min indicating a very slow turn over of PML V molecules at nuclear bodies (Fig. 3E). This observation corroborates our previous conclusion that PML V may act as a hyper-stable scaffold component within PML nuclear bodies [25].

Human cells contain six nuclear PML isoforms, whereas for mouse cells only two PML transcripts have been described so far, and the corrsponding mouse PML sequences are more than 80% similar to human PML isoform I [29]. It should therefore be pointed out that PML isoforms II to VI have no direct counterparts in the murine system with respect to alternative expression of exons 6 to 9. Nevertheless, the analysis of these human isoforms in mouse cells may still deliver valuable information on PML nuclear body formation, particularly in a PML-negative background. Based on the high sequence similarity the human GFP-PML I construct has the potential to functionally (and thus dynamically) act in a similar fashion as mouse PML in murine cells. Indeed, the exchange dynamics of human GFP-PML I protein at nuclear bodies of PML-positive murine cells was similar to human cells (Fig. 3A) [25]. In contrast, GFP-PML I was alomst immobile at nuclear bodies in 3T3 cells lacking endogenous PML (Fig. 3A). The same phenomenon was observed for GFP-PML isoform III and in a subpopulation of cells expressing GFP-PML isoforms II or IV (Fig. 3, B-D). Thus, in the absence of endogenous PML proteins, GFP-PML I to IV form stable aggregates. These observations suggest that these isoforms require the presence of other PML isoforms or endogenous PML-binding proteins for efficient exchange at nuclear bodies. They also suggest that the capacity to contribute to nuclear body stability is inherent to all of these PML isoforms. We also observed a minor population (< 20%) of cells in which GFP-PML isoforms II and IV showed dynamic exchange at nuclear bodies (Fig. 3B, and 3D, red curves). These observations suggest a cell cycle dependent behavior of PML II and PML IV at nuclear bodies which likely originate from interaction of these isoforms with as yet unknown binding sites outside nuclear bodies. However, since human PML II and PML IV are not conserved in mouse cells, such interactions might be biologically non-relevant. In contrast to GFP-PML I to IV, the dynamics of GFP-PML V and VI were almost unaltered in PML negative cells (Fig. 3E, and 3F).

The presence of slow exchanging populations of GFP-PML I to IV is consistent with the idea that these isoforms are also able to provide nuclear body stability, as concluded for PML V. In human cells, GFP-PML I and III exhibit much higher exchange rates at nuclear bodies [25] than observed here in mouse cells (Fig. 3).

That GFP-PML I and III do not exchange with soluble nucleoplasmic populations in mouse cells may indicate that the human isoforms can not be dissociated from nuclear bodies through interaction with soluble mouse PML-binding proteins outside nuclear bodies. Since the SUMOylation status of PML also regulates the exchange rate at nuclear bodies [25], changing SUMO patterns on GFP-PML I to IV may also explain their changing exchange rate at NBs.

### 3.3 A kinetic model to quantitatively describe PML nuclear body assembly

**Figure 4:**
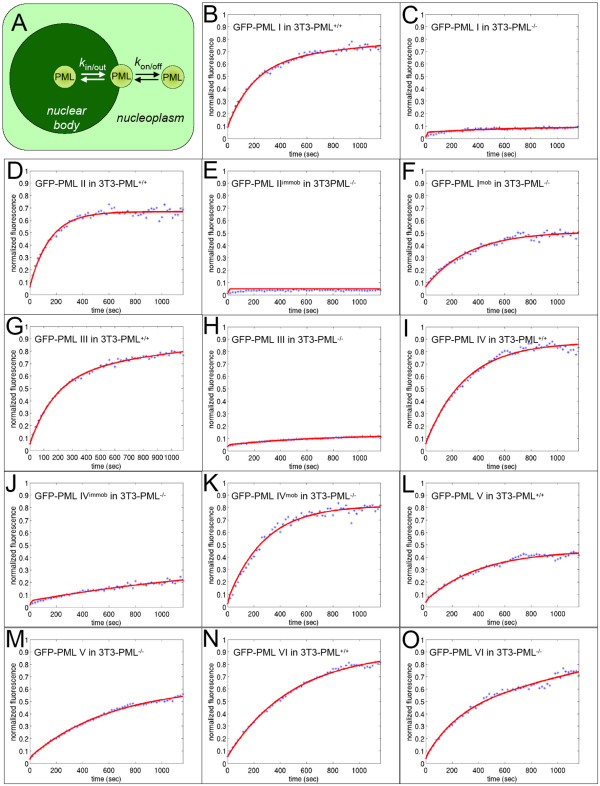
**Kinetic model for molecule exchange at PML nuclear bodies**. (A) Molecules with the potential to interact with PML nuclear bodies move by diffusion in the nucleoplasm outside nuclear bodies. Upon stochastic encounter, molecules associate and dissociate from the periphery of the nuclear body (*k*_on _and *k*_off_, respectively) and penetrate into and out of the shell of the nuclear body (*k*_in _and *k*_out_, respectively). (B-O) Fitting of the measured FRAP curves (blue dotted line) with the binding-diffusion model shown in (A) results in good fits (red lines).

In order to understand PML nuclear body assembly in a more quantitative way we applied a kinetic modeling approach established previously [25] (Fig. 4A). Diffusion inside and out of bleached regions was modelled as a linear two-way process as described in the materials and methods section. The diffusion coefficients of the GFP-PML isoforms, as determined by fluorescence correlation spectroscopy, ranged between D = 1 - 3 μm^2^s^-1 ^(data not shown). Table 1 contains for each of the PML protein isoforms the binding and unbinding rate *k*_on _and *k*_off_, and the rate of movement to the inner core and to the outer surface of the body, *k*_in _and *k*_out_. From these, we computed the residence time (R.t.), i.e. the mean time a molecule spends bound to the nuclear body, and the fraction of molecules bound in the inner and outer region of the body, bnd_in _and bnd_out_. This model provided good fits to the measured FRAP curves of all PML isoforms (Fig. 4, B-O). This quantitative evaluation confirmed that GFP-PML V had the longest residence time (947 s) of all isoforms in PML-positive cells (Table 1). The residence times of GFP-PML isoforms I to IV ranged between 221 and 336 seconds which is very similar to their residence times at nuclear bodies in human cells [25]. Thus, each PML isoform exhibits individual exchange characteristcs at nuclear bodies. Since the molecular structure of PML isoforms are identical over two-thirds of the sequence at the N-termiuns, the observed differences in the dynamic behaviour must originate from their C-terminal varying parts.

**Table 1:** Exchange dynamics of PML isoforms at nuclear bodies

Protein	Cells	*k*_on_(s^-1^)	*k*_off_(s^-1^)	*k*_in_(s^-1^)	*k*_out_(s^-1^)	R.t. (s)	bnd_out_	bnd_in_
GFP-PML I	PML^+/+^	0.0633	0.0051	0.0002	0.0004	300	0.65	0.35
GFP-PML I	PML^-/-^	0.0015	0.0015	>0.0001	0.0049	648	1.00	0.00
GFP-PML II	PML^+/+^	0.0856	0.0069	>0.0001	>0.0001	222	0.65	0.35
GFP-PML II^immob^	PML^-/-^	0.0000	n.d.	n.d.	n.d.	n.d.	n.d.	n.d.
GFP-PML II^mob^	PML^-/-^	0.0288	0.0031	>0.0001	>0.0001	659	0.49	0.51
GFP-PML III	PML^+/+^	0.0692	0.0055	>0.0001	0.0007	274	0.67	0.33
GFP-PML III	PML^-/-^	0.0023	0.0013	>0.0001	0.5592	744	1.00	0.00
GFP-PML IV	PML^+/+^	0.0565	0.0034	>0.0001	>0.0001	337	0.87	0.13
GFP-PML IV^immob^	PML^-/-^	0.0040	0.0005	>0.0001	>0.0001	4777	0.38	0.62
GFP-PML IV^mob^	PML^-/-^	0.0571	0.0037	>0.0001	>0.0001	333	0.81	0.19
GFP-PML V	PML^+/+^	0.0199	0.0024	>0.0001	>0.0001	957	0.43	0.57
GFP-PML V	PML^-/-^	0.0188	0.0016	>0.0001	>0.0001	1010	0.61	0.39
GFP-PML VI	PML^+/+^	0.0351	0.0021	>0.0001	>0.0001	541	0.90	0.10
GFP-PML VI	PML^-/-^	0.0385	0.0036	0.0008	0.0010	493	0.56	0.44

The diffusion-binding model described in Figure 4A was used to extract kinetic data from the FRAP and FCS experiments for all PML isoforms in PML positive and PML negative cells. *k*_on_: association rate at the surface of the nuclear body; *k*_off_: dissociation rate at the surface of the nuclear body; *k*_in_: penetration rate into the nuclear body; *k*_out_: penetration rate out of the core of the nuclear body; R.t.: mean residence time at nuclear bodies; bnd_out_: fraction of molecules residing at the surface of the nuclear body; bnd_in_: fraction of molecules residing in the core of the nuclear body.

Compared to PML-positive cells, the residence time at nuclear bodies of GFP-tagged PML isoforms I to IV was increased several-fold in PML-negative cells (Table 1). This result suggests that the exchange rate of overexpressed human PML isoforms is influenced by the dynamics of the endogenous mouse PML proteins. Thus, in the absence of endogenous mouse PML protein, the unrelated human isoforms tend to form more insoluble aggregates. Interestingly, this is not true for the shortest PML isoforms V and VI, the residence time of which is almost identical independent of the presence or absence of endogenous PML bodies (Table 1). This individual property of GFP-PML V and VI argues in favor of a more structural role for these isoforms at PML nuclear bodies.

### 3.4 SUMOylation of PML is required to form nuclear bodies

**Figure 5:**
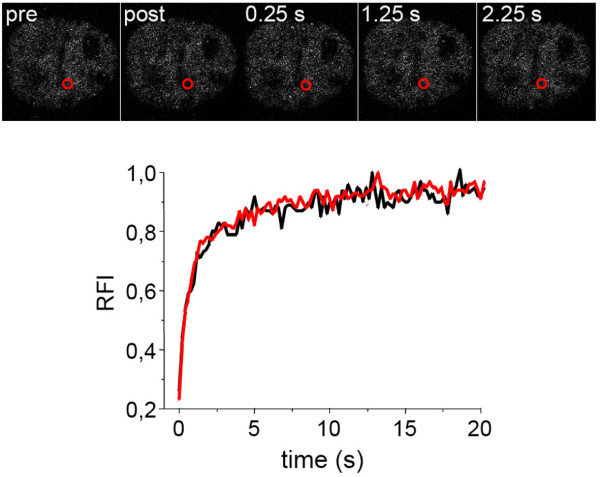
**PML SUMOylation sites K160 and K490 are required for PML body formation**. The upper part shows a FRAP experiment of GFP-tagged mutant PML IV (K160/490R) in 3T3-PML^-/- ^cells. Fluorescence was bleached in a circled area within the nucleoplasm (red circle). Confocal images were taken before (pre) and after (post) the bleach pulse and at different time points thereafter. Bottom part: FRAP experiments of GFP-tagged PML IV (K160/490R) were performed in 3T3-PML^+/+ ^(black) and 3T3-PML^-/- ^cells (red) and quantified. FRAP curves show mean values of at least ten measurements.

PML contains three Lysine residues (K65, K160, and K490; Fig. 1A) at which all isoforms can be SUMOylated in vivo [30]. To determine the impact of SUMO modifications we analyzed the localization and dynamics of a GFP-PML IV construct in which K160 and K490 were mutated to Arginine. This mutant protein localized diffusely in the nucleoplasm of both PML-positive and PML-negative cells (Fig. 5, and data not shown) indicating that SUMOylation at K160 and K490 are required for PML nuclear body binding. FRAP analysis of the SUMO mutant within a nucleoplasmic region revealed fast and complete recovery within seconds suggesting a predominant diffusion type of mobility (Fig. 5). We had shown previously that the K160/490R mutant is still able to bind to nuclear bodies in human cells but its residence time was only 5.8 s compared to the residence time of wild-type GFP-PML IV, which is 7.6 min [25]. Thus, the human K160/490R mutant very transiently interacts at 'human' PML bodies [25] but does not bind to 'mouse' PML bodies and is not able to form nuclear body structures in the absence of any PML protein (Fig. 5). Bacterially expressed PML protein is not SUMO-modified but still able to form nuclear body-like structures *in vitro *through self-assembly of the N-terminal RBCC region of PML [31]. Because GFP-PML-K160/490R is not able to form nuclear body structures in the absence of other mouse or human PML proteins (Fig. 5) we conclude that SUMOylation is a critical determinant for PML body formation. In future studies it will be intersting to analyze the impact of single, double and the triple SUMO mutants of PML in nuclear body formation.

### 3.5 Assembly properties of PML bodies are different from other subnuclear domains

**Figure 6:**
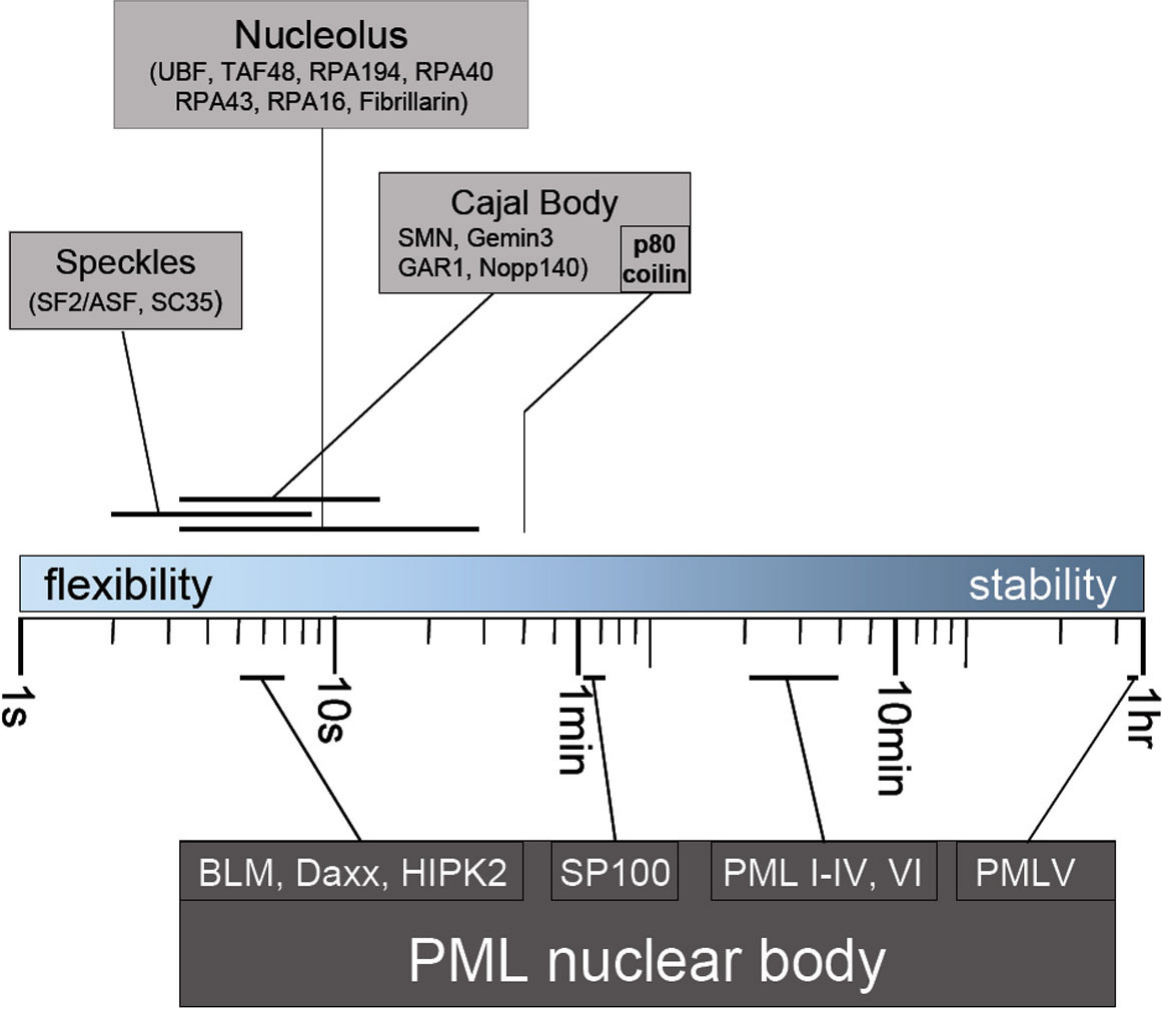
**Assembly dynamics of subnuclear domains**. The residence times of components of the indicated subnuclear domains are depicted on a logarithmic scale between 1 second and 1 hour. Fast or slow exchanging components provide flexibility or stabilty, respectively, probably in terms of both structue and function of subnuclear domains. Residence times of speckle, nucleolus and Cajal body compoents were assessed from FRAP experiments published previously [32-39]. Residence times of PML nuclear body components were taken from [25].

FRAP analyses of subnuclear domains such as speckles, Cajal bodies and nucleoli had revealed that their component parts rapidly exchange with nucleoplasmic pools [32-39]. Typical residence times of proteins within these compartments are in the seconds range (Fig. 6). These observations have led to the conclusion that nuclear body proteins undergo repeated and rapid cycles of association and dissociation between the nuclear body and the nucleoplasm [40]. As a consequence, a nuclear body is in perpetual flux and its structure is determined by the ratio of on-rate versus off-rate of its components [41]. While this assembly mechanism is certainly true for speckles, Cajal bodies and nucleoli, the work presented here and previously [25] demonstrate that the stabilty of PML nuclear bodies relies on very long residence times of specific PML isoforms, in particular PML V [25] (Fig. 6), and probably also PML VI (Table 1). These observations strongly support the idea of a scaffold function of PML nuclear bodies [42].

## 4 Conclusions

Four main mechanisms are known through which cellular scaffolds can modify signalling between active components [43]. They can (i) tether enzymes close in space and enhance effective local concentrations, (ii) mediate assembly of signalling complexes in a combinatorial manner, (iii) dynamically regulate turnover or accessibility of specific factors, or (iv) modify the conformation of enzymes binding to them [43]. All these potential functions are fully compatible with the biophysical properties of PML nuclear bodies assessed in this report and previously [25]. The scaffold model for PML body function is also compatible with the biochemistry (phosphorylation, SUMOylation, acetylation) believed to occur on specific nuclear proteins at these macromolecular assemblies [8]. Interestingly, although direct evidence is lacking so far, PML NBs have recently been suggested as scaffolds for caspase-2 mediated cell death [44]. Future research should therefore aim to establish new experimental approaches with which the potential function of PML nuclear bodies as nuclear scaffolds can be tested in a more direct and functional way.

## Authors' contributions

PB and PH acquired the experimental data. TL performed mathematic modeling of the experimental data. Data were analyzed and interpreted by PB, TL and PH. PH wrote the manuscript.
